# Molecular Mechanisms of MmpL3 Function and Inhibition

**DOI:** 10.1089/mdr.2021.0424

**Published:** 2023-05-04

**Authors:** John T. Williams, Robert B. Abramovitch

**Affiliations:** Department of Microbiology and Molecular Genetics, Michigan State University, East Lansing, Michigan, USA.

**Keywords:** *Mycobacterium tuberculosis*, MmpL3, phenotypic drug discovery

## Abstract

species include a large number of pathogenic organisms such as *Mycobacterium tuberculosis*, *Mycobacterium leprae*, and various non-tuberculous mycobacteria. Mycobacterial membrane protein large 3 (MmpL3) is an essential mycolic acid and lipid transporter required for growth and cell viability. In the last decade, numerous studies have characterized MmpL3 with respect to protein function, localization, regulation, and substrate/inhibitor interactions. This review summarizes new findings in the field and seeks to assess future areas of research in our rapidly expanding understanding of MmpL3 as a drug target. An atlas of known MmpL3 mutations that provide resistance to inhibitors is presented, which maps amino acid substitutions to specific structural domains of MmpL3. In addition, chemical features of distinct classes of Mmpl3 inhibitors are compared to provide insights into shared and unique features of varied MmpL3 inhibitors.

## Introduction

*Mycobacterium tuberculosis* (Mtb) is the bacterium that causes tuberculosis (TB) in humans. In 2020, the WHO estimated that 10 million people became sick with TB and 1.5 million people died from the disease.^1^ Currently, no vaccine protects against pulmonary TB.^1^ In the absence of an effective vaccine, antibiotic therapy requires patients to take a daily combination of four drugs, including rifampin (RIF), isoniazid (INH), ethambutol (EMB), and pyrazinamide, for 6 months. However, the long course of treatment and incomplete therapy have led to the selection and evolution of multidrug-resistant (MDR) and extensively drug-resistant (XDR) Mtb strains, which are currently spreading person to person.^2^ Therefore, additional therapeutic targets and strategies need to be identified. In addition, other pathogenic non-tuberculous mycobacteria (NTM), such as *Mycobacterium avium* complex (MAC) and *Mycobacterium abscessus* (MAB), are emerging as common causes of infections, particularly in the immunocompromised, the elderly, and those with predisposing conditions, such as cystic fibrosis. These NTMs are resistant to most Mtb drugs^3,4^ and therefore new drugs are also needed to control NTM-mediated diseases.^5^

Over the last few decades, high-throughput screens (HTS) were conducted to identify the next Mtb drug. Subsequent studies into the mechanism of action of hits identified from these screens have identified QcrB,^6–14^ DprE1,^15–20^ and mycobacterial membrane protein Large 3 (MmpL3)^20–37^ as recurring targets. Of these three targets, the essential mycolic acid (MA) flippase MmpL3^20–37^ is the most commonly identified with 18 studies reporting over 30 chemical scaffolds targeting MmpL3.^20–38^ While several of the MmpL3 inhibitors have overlapping chemical groups (discussed further below), the specific structural differences between them make predicting MmpL3 inhibitors within a chemical library difficult. However, the essential nature of MmpL3 makes it a highly sought-after therapeutic target for Mtb.^39^ Owing to its high therapeutic potential, several studies over the last decade have increased our understanding of MmpL3 in terms of function, regulation, and protein-substrate/inhibitor interactions. This review will discuss the function of MmpL3 in mycobacteria as well as the molecular insights gained from studying MmpL3 and the inhibitors proposed to target this essential flippase.

## Synthesis and Transport of TMM and TDM

**FIG. 1.**
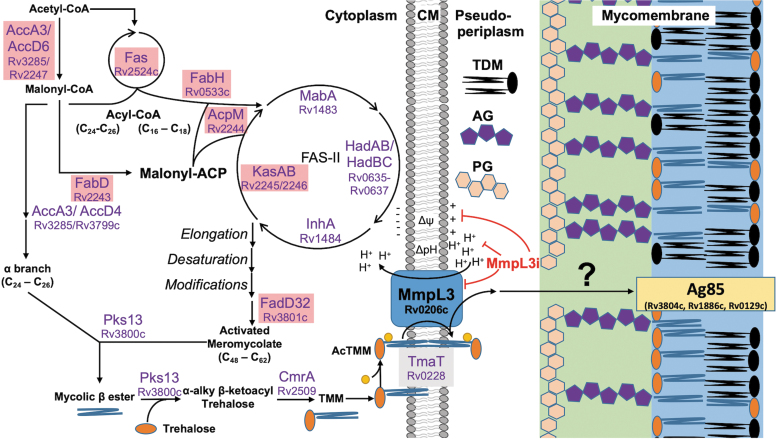
The biosynthetic pathway of TMM and TDM. The illustration of the biosynthetic route of TMM and TDM in mycobacteria. Gene names of each step include corresponding gene numbers from *Mycobacterium tuberculosis* H37Rv. Enzymes highlighted in *red* are genes that are downregulated following MmpL3 disruption. Δψ, membrane potential; AG, arabinogalactan; CM, cytoplasmic membrane; MmpL3i, unspecified MmpL3 inhibitor; PG, peptidoglycan; *yellow* sphere attached to TMM is an acetyl group.?—an unknown transport system that shuttles TMM to the mycomembrane. MmpL3, mycobacterial membrane protein large 3; TDM, trehalose dimycolate; TMM, trehalose monomycolate.

**FIG. 2.**
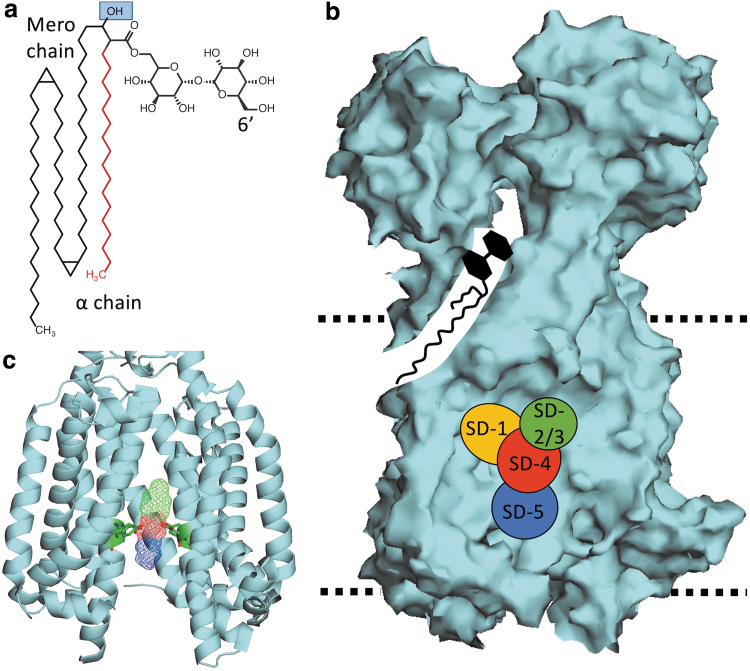
TMM and MmpL3 Structures. **(a)** The structure of a simple TMM. The α-chain is illustrated in *red*; the *blue square* indicates the site of acetylation by TmaT. The site of the acyl transferase reaction carried out by the Ag85 complex, 6′C, is also labeled. **(b)** A surface illustration of MmpL3 (PDB: 6AJH). The illustration shows a simple model of TMM shuttling form the CM toward the periplasmic porter domains. The binding domains SD-1 to SD-5 are superimposed onto this model. The *dotted lines* indicate the approximate position of the CM. **(c)** An illustration of the transmembrane region of MmpL3 (PDB: 6JAH) bound to a model MmpL3 inhibitor shown in wire form. TMD 10 has been removed to show binding of the model inhibitor. The amino acids illustrated show the Tyr-Asp pairs required for proton relay. PDB, protein data bank.

MmpL3 is the flippase and the sole transporter for the essential branched long chain (C_60–90_) glycolipid TMM (illustrated in Fig. 2a) synthesized in the cytoplasm or cytoplasmic membrane (CM). The complete synthetic pathway of TMM is still not fully understood and some differences exist between species of mycobacteria. An overview of the TMM synthetic pathway is outlined in Fig. 1 and a description is as follows: acetyl-CoA (C_2_) and malonyl-CoA (C_3_) generated from catabolic pathways serve as primers for the synthesis of short chain (C_24–26_ or C_16–18_) fatty acids (FAs) by the eukaryotic like fatty acid synthase (FAS)-I enzyme Fas (Rv2524c).^40,41^ From here, MA synthesis diverges along two paths to form the α-branch and the meromycolate chain.

The α-branch consists of carboxyacyl-CoA (C_24–26_) (Fig. 2a, red) and is formed by the acyl-CoA carboxylase complex consisting of AccA3 (Rv3285) and AccD4 (Rv3799c).^42^ Along the other branch, C_16–18_ short chain FAs are elongated by the β-ketoacyl acyl carrier protein (ACP) synthase mtFabH (Rv0533c), which condenses a malonyl substrate, carried by the ACP AcpM (Rv2244), to form β-keto-thioester.^43–45^ Malonyl-AcpM is formed by the transfer of malonyl from malonyl-CoA to AcpM by the enzyme mtFabD (Rv2543).^46–48^ The FAS-II system is composed of several enzymes, including the β-ketoacyl-reductase MabA (Rv1483),^49–52^ the β-hydroxyacyl-ACP dehydratases HadAB/HadBC (Rv0635-0637),^53,54^ the *trans*-2-enoyl-ACP reductase InhA (Rv1484),^55–57^ and β-ketoacyl-ACP synthases KasA/KasB (Rv2245/Rv2246).^58,59^

It is hypothesized that to form the fully mature long chain FAs, up to three unique FAS-II complexes exist and are composed of specific combinations of the FAS-II enzymes.^60,61^ From here, long chain FAs undergo several reactions independent of the FAS-II complex, including modifications, such as desaturation and cyclopropanation,^62–69^ to form the meromycolate chain (C_48–62_). FadD32 (Rv3801c) then activates the meromycolate chain^42,70–72^ to undergo a multistep reaction carried out by the polyketide synthase Pks13 (Rv3800c). Pks13 induces a condensation reaction between the meromycolate chain and the α-branch to form mycolic β-ketoesters.^70,73^ Pks13 then carries out an additional reaction to add the disaccharide trehalose to the β-ketoester to form the α-alkyl β-ketoacyl trehalose glycolipid.^74^ In the final synthesis step, CmrA (Rv2509) reduces α-alkyl β-ketoacyl trehalose to form the mature MA TMM (Fig. 2a).^75^

Several steps in the synthesis of TMM are still not understood, including double bond formation in MAs. In this review, we illustrate MA modifications such as cyclopropanation occurring after exiting the FAS-II complex; however, when exactly these steps occur is unknown and may occur after TMM is transported into the CM. In addition, not all TMM synthetic pathways are the same and can result in different chain lengths and structural modifications.^76,77^ For example, TMM of Mtb includes cyclopropyl, keto-, methoxy, and hydroxyl groups, while TMM of *Mycobacterium smegmatis* contains *cis*-, *trans*-, or epoxy groups.^77^ Furthermore, both meromycolate and α-chain length can vary between species.^78^ Chain length regulation is also not fully understood, but is likely regulated by higher order protein-protein and protein-cofactor interactions.^40,79,80^ TMM is essential for mycobacteria cell viability, and therefore many proteins involved in the synthesis of TMM are also essential.^81–85^ Consistent with a model of essentiality, enzymes involved in MA synthesis are often identified as targets of anti-TB inhibitors. Notable targets include InhA and HadAB, which are inhibited by the TB drugs INH^56^/ethionamide^86,87^ and isoxyl^88^/thiacetazone,^88^ respectively.

The above section describes the synthesis of TMM, which has been fairly well characterized over the last half century. However, only recent efforts have managed to characterize the flipping of TMM across the CM, but some gaps do still exist. One essential step required before transport by MmpL3 is that TMM must first be acetylated (acTMM) by TmaT (Rv0228).^89,90^ TmaT is an essential integral membrane protein^82,83,85,89^ and it is hypothesized that TmaT plays a role in intercalation of (ac)TMM into the inner leaflet of the CM.^90^ However, how this occurs is unclear and additional enzymes may be involved in this step. For example, two recent studies identified a putative methyltransferase MtrP (NCgl2764, Rv0224c) and the membrane protein MmpA (NCgl2761, Rv0226c) as being required for efficient transport of the *Corynebacterium* TMM equivalent trehalose monohydroxycorynomycolate from the CM to outermembrane in *Corynebacterium glutamicum*.^91,92^ Both of these genes are conserved in mycobacteria, and were identified as essential based on transposon mutagenesis studies^82,83,85,89^; however, their functions have yet to be studied in mycobacteria. Also, TtfA (Rv0383c), a protein of unknown function, was demonstrated to interact with MmpL3 and is required for TMM transport, but its role in TMM transport is still unknown.^93^ Taken together, the identification of three essential proteins for (ac)TMM transport suggests that MmpL3 alone may not be the sole protein involved in MmpL3 flipping.

Following full maturation and acetylation, TMM is then transported across the CM by the dedicated flippase MmpL3 (Rv0206c). This flipping action occurs in a two-step process powered by membrane energetics (discussed further in the next section). Following flipping, (ac)TMM is associated with the porter domains of MmpL3, where it is hypothesized that (ac)TMM is handed off to a yet identified chaperone system to be transported across the peptidoglycan (PG) and arabinogalactan (AG) layers to the mycomembrane (MM) (Fig. 1). Once in the MM, TMM can either accumulate or undergo two different acyltransferase reactions carried out by the Ag85 complex (FbpA, FbpB, and FbpC2).^94–98^ The first acyltransferase reaction removes the trehalose moiety and covalently links the MA to the terminus of the AG layer forming mycolyl-arabinogalactan-peptidoglycan (mAGP), a single molecule covalently linking the PG, AG, and MM.^96^ This acyltransferase reaction is essential for cell viability,^96,97^ and is the reason why MmpL3, as the sole transporter of TMM, is essential. The second possible acyltransferase reaction moves the MA moiety of one TMM molecule to the 6′C of a second TMM molecule to form trehalose dimycolate (TDM) (Fig. 2a).^96^ TDM is also known as “Cord Factor” due its role in the formation of mycobacterial cords,^99^ a multicellular aggregate characteristic of mycobacteria.^100^ TDM plays roles as a protective barrier,^69,97,101^ biofilm formation,^102^ granuloma formation,^103–108^ and macrophage stimulation.^106–109^ While mAGP faces the periplasm space, the localization of TDM in the inner or outer leaflet of the MM following synthesis is not clear. Future studies may seek to understand the (a)symmetry of TDM across the MM.

Preceding the acyltransferase reactions in the MM, several questions revolving around the fate of acTMM following transport across the CM still remain. For one, it is still unclear when TMM undergoes de-acetylation (acTMM to TMM) and whether or not this is an essential step. Second, one of the main pressing questions concerning the fate of (ac)TMM is how it is transported across the PG and AG layers. Surface plasmon resonance (SPR) data from Belardinelli et al demonstrated that MmpL3 does not directly interact with proteins from the Ag85 complex.^110^ This is consistent with distance measurements that the 35 Å porter domains protruding from the CM would not reach across the 30–40 nm gap between the CM and the MM.^110–112^ Two studies have attempted to find periplasmic interacting partners of MmpL3 using the bacterial adenylate cyclase-based two-hybrid (BACTH) system and protein co-precipitation methods, but neither identified any periplasmic or cell wall localized candidates.^93,110^ Further studies into (ac)TMM transport may identify additional drug targets, as well as further our understanding of the physiology and cell wall biogenesis of mycobacteria.

## MmpL3 Protein Structure and Function

MmpL3 (illustrated in Fig. 2b) is one of several MmpL proteins found in mycobacteria^113^ and is a member of the resistance nodulation and division (RND) superfamily of proteins.^89,114–121^ MmpL3 has three main structural regions, a transmembrane region that spans the CM, a porter domain consisting of two large loops in the periplasmic space, and a cytoplasmic C-terminal domain. The transmembrane region is made up of 12 transmembrane α-helix domains (TMD 1–12).^89,116,117,120–122^

Like other RND proteins, MmpL3 is powered by proton translocation through a central vestibule made up of TMDs 4, 5, 6, 10, 11, and 12.^89,123^ Proton translocation is guided by conserved Asp/Tyr pairs on TMD 4 and 10 (Fig. 2c).^89,115,117,119–122^ Furthermore, the transmembrane region also serves to interact with lipid substrates. Recently, TMD 7–10 were shown to play a role in lipid binding based on cryo-EM structures of TMM bound to MmpL3_Mtb._^124^ The 12 TMDs are conserved among the 13 MmpL proteins of Mtb, with the exception of MmpL13, which seems to have undergone a genetic cleavage event in which the protein was split into 2 genes (*mmpL13a/Rv1145* and *mmpL13b/Rv1146*).^125^

While the domain architecture of MmpL3 is similar to the other 12 MmpL proteins in Mtb,^116^ one key difference is the presence of a large cytoplasmic C-terminal domain, found only in MmpLs 3, 11, and 13^114,116^ and rarely found in canonical RND proteins.^114,116^ The C-terminal domain is involved in protein localization,^110^ protein-protein interactions,^93,110^ and post-translational phosphoregulation (all discussed further below).^126,127^ This C-terminal domain is nonessential^89^ and is not found in other canonical RND proteins.^114,116^ The tertiary structure of MmpL3 from *M. smegmatis* (MmpL3_Msm_) has been resolved and crystal structures have suggested that MmpL3 is a monomer.^120–122^ However, these structures were from truncated forms of MmpL3 lacking the C-terminal domain.^120–122^ Recently, a BACTH system study suggested that full-length MmpL3 forms an oligomer.^110^ A complexed MmpL3 is consistent with previous modeling predictions^89^ and observations for canonical RND proteins such as AcrB of *Escherichia coli*.^123^

Interestingly, full-length MmpL3 from either *M. smegmatis* or Mtb has been difficult to purify^120,121,124,128^ and the model confidence in the predicted AlphaFold structure of MmpL3 drops dramatically for the cytoplasmic domain.^129,130^ A brief analysis using the Intrinsically Unstructured Protein Prediction server (IUPred2A)^131,132^ suggests that the C-terminal domain is highly disordered (Supplementary Fig. S1), which would explain the inability to purify this domain of the protein. Alternatively, post-translation modification, such as phosphorylation (discussed in a separate section) may be required to stabilize the structure before purification.

The structure of MmpL3 is further characterized by two large periplasmic porter domains located between TMD 1 + 2 and 5 + 6.^89,114,120–122^ These porter domains were demonstrated to interact with the lipids phosphatidylethanolamine^120^ and TMM.^124^ Purified porter domains of MmpL3 were also shown to interact with heme,^133^ but it is unclear what role MmpL3 may play in iron metabolism. It is hypothesized that these porter domains play a role in handing off exported TMM to the aforementioned unidentified chaperone system to transport TMM to the MM; however, other proteins could also be involved in this process.

As mentioned above, following acetylation, acTMM is flipped across the CM in a two-step mechanism. The first step relies on the proton motive force (PMF), which flips acTMM from the inner to the outer leaflet of the CM (Fig. 1). The evidence for this first step primarily comes from a spheroplast assay developed by Xu et al. This assay measures the abundance of (ac)TMM that is fluorescently labeled once deposited into the outer leaflet of *M. smegmatis* spheroplasts.^117^ Xu et al observed that labeled (ac)TMM accumulates in the outer leaflet in untreated cells; however, following treatment with MmpL3 inhibitors, such as AU1235 or BM212, or PMF uncouplers, such as CCCP or nigericin, fluorescence is diminished.^117^ These data clearly demonstrated that MmpL3 is involved in (ac)TMM flipping across the CM and that (ac)TMM transport may be a two-step mechanism. The second step in (ac)TMM flipping and transport involves MmpL3 shuttling (ac)TMM from the outer leaflet of the CM to be transported to the MM. This step is carried out by TMDs 7–10, which seem to relay TMM to the porter domains. Evidence for this primarily comes from a recently published cryo-EM study by Su et al, which captured TMM in both the transmembrane domain and the porter domain (Fig. 2b).^124^ This study was remarkably informative in how MmpL3 transports (ac)TMM from the CM to the porter domains, which are then hypothesized to hand (ac)TMM off to an unidentified chaperone system, as described above.

Taken together, the results of the spheroplast assay and cryo-EM structures generally agree with a two-step model that was predicted, but never demonstrated. While these studies have elucidated much of the mechanism that drives TMM flipping, two important questions remain unanswered. The first is how MmpL3 recognizes acTMM before flipping and what protein domains drive flipping from the inner to the outer leaflet of the CM. A recent photoactivatable probe was created by Kavunja et al that structurally resembles TMM.^134^ Upon photoactivation, this probe is covalently linked to interacting proteins and allows for enrichment of proteins that associate with the probe. This probe enriched several proteins in *M. smegmatis* cells previously demonstrated to interact with TMM, including MmpL3. The authors identified the enriched proteins through peptide-based MS; however, which residues the probe specifically linked to were either not identified or not reported. Additional experiments using these probes with pre-enriched MmpL3-expressing cells or inverted membrane vesicles may identify which MmpL3 residues TMM interacts with in the inner leaflet of the CM.

The second unanswered question is following incorporation of acTMM to the porter domain, how is acTMM handed off to the next protein and which protein(s) is/are responsible for chaperoning acTMM to the MM. Several studies have attempted to identify periplasmic MmpL3 interactors; however, neither reported any major hit.

Furthermore, while this review and many studies have focused on the role of MmpL3 in TMM transportation, a recent study reported that MmpL3 interacts with additional lipids, including cardiolipin, diacylglycerol, phosphatidylglycerol, and phosphatidylinositol.^120^ More importantly, this study also reported co-crystal structure of the lipid phosphatidylethanolamine bound to the porter region of MmpL3 in a way that was highly similar to what was reported for TMM. This suggests that MmpL3 is not solely dedicated to TMM transport; however, the biological consequences of these findings have yet to be investigated.

The number of *mmpL* genes varies between mycobacteria. For example, *Mycobacterium leprae* encodes only 5 *mmpL* genes, while Mtb encodes 13 *mmpL* genes, and *Mycobacterium immunogenum*, of the MAB complex, encodes 29 *mmpL* genes.^116^ However, only *mmpL3* and *mmpL11* are conserved in all species of mycobacteria^116^ and only *mmpL3* is essential for growth and viability both *in vitro* and *in vivo* in most mycobacteria.^29,81–85,89,135–140^

Comparative protein structure prediction studies show that Mtb MmpL proteins fall into two distinct clusters.^114^ MmpL3 is a member of the Cluster II MmpL proteins, along with MmpL11 and MmpL13, with the remaining 10 Mtb MmpL proteins falling into Cluster I. Cluster II MmpL proteins are distinguished from Cluster I MmpL proteins by the inclusion of the cytosolic C-terminal domain and the lack of a docking domain in the second porter loop.^114^ It should be noted that some MmpL proteins have associated mycobacterial membrane protein small (MmpS) proteins, which are typically encoded adjacent to their cognate *mmpL* genes.^113^ MmpS proteins have been hypothesized to help in MmpL function, although how they do this is not clear. A study looking into the role of the semiredundant MmpS4 and MmpS5 proteins indicates that they play a role in assisting the cognate MmpL4 and MmpL5 proteins in iron scavenging.^141^ In addition, not all MmpL proteins have associated MmpS proteins, including MmpL3.^142^ While the exact function of all MmpL proteins in mycobacteria is not yet clearly defined, it is largely believed that they serve as substrate exporters, including xenobiotic efflux.^143^

## MmpL3 Localization and Interactome

Incorporation of TMM into the cell wall of mycobacteria during cell division is an essential process. Mycobacteria undergo asymmetric cell division and extend from the old pole.^144^ Therefore, it stands to reason that proteins involved in cell wall synthesis, including MmpL3, would localize to the same area. Indeed, fluorescently labeled MmpL3 was demonstrated to localize at the dividing pole during cell division.^33,93,110,118,145,146^ Localization of MmpL3 to the dividing pole is guided, in part, by the C-terminal domain, as mycobacteria expressing truncated MmpL3 lacking the C-terminal domain show decreased MmpL3 polar localization during cell division.^110^ In addition to the C-terminal domain, the DivIVA homolog Wag31 (Rv2145c) may also play a role in MmpL3 localization.^147^ While it is not clear if Wag31 directly interacts with MmpL3,^147^ Wag31 does coordinate cell division machinery to the pole during cell division, including enzymes involved in PG synthesis^148,149^ and MA biosynthesis, such as AccA3.^147^

Recent reports have identified additional proteins that directly interact with MmpL3, including additional proteins involved in MA transport, as well as proteins involved in PG and AG substrate transport.^93,110^ MmpL3-interacting proteins involved in TMM transport included the TMM acetylation protein TmaT,^110^ as well as TtfA (Rv0383c) and Msmeg_5308 (Rv1057).^93^ The function of TtfA is not fully defined, but is essential in mycobacteria and is required for the transport of TMM,^93^ while MSMEG_5308 is not required for TMM transport and dispensable for cell viability,^150^ but may stabilize TtfA-MmpL3 interactions during cell stress.^93^ MmpL3 was also demonstrated to interact with MmpL11 (Rv0202c),^110^ which is involved in the transport of MA-containing lipids, monomeromycolic diacylglycerol, and mycolate wax ester.^151^

Additional proteins of unknown or ill-defined function were also identified as possible MmpL3 interactors based on a BACTH screen, including Rv0204c, Rv0207c, Rv0625c, Rv1275 (LprC), Rv1337, Rv1457c, Rv1799 (LppT), Rv2169c, Rv3064c, Rv3271c, Rv3483c, Rv3909, and MT2653,^110^ which may allude to the existence of a large cell wall transport complex; however, these hits have yet to be verified using alternative methods. Notably MmpL3 does not interact with the Ag85 complex or enzymes involved in MA synthesis enzymes such as FAS-II enzymes or Pks13.^93,110^ While a lack of interaction between the Ag85 complex and MmpL3 could be predicted based on distance measurements as discussed above,^110^ the lack of interaction between MA synthesis enzymes and MmpL3 was surprising, given they are co-coordinated by Wag31^147^ to localize to the dividing pole^146^ and are co-regulated by PknAB/PstP.^126,127,148^

Current studies of MmpL3 localization and co-localization have focused on dividing cells to gain better insights into cell wall biogenesis and the mycobacterial divisome.^33,93,110,118,145,146^ However, questions remain for where MmpL3, and other divisome proteins, localizes during states of nonreplication when MmpL3 is dispensable for cell viability,^89,137^ and some MmpL3 inhibitors do not kill nonreplicating mycobacteria.^22,152^ Using scanning electron microscopy, Lun et al observed that Mtb treated with an MmpL3 inhibitor developed dimples at the dividing pole where MmpL3 localizes.^153^ They hypothesized that these dimples were actually holes forming at the site of MmpL3 localization leading to cell death.^153^ If MmpL3 does not localize to the dividing pole during states of nonreplication, these holes may not develop, which could explain why MmpL3 inhibitors do not kill nonreplicating cells. Future studies may seek to address where proteins involved in cell wall synthesis and division, including MmpL3, localize during states of nonreplication.

## Regulation of MmpL3

*mmpL3* is encoded in a monocistronic operon in mycobacteria.^136^ To date, the transcriptional regulation of *mmpL3* is not fully understood. However, Chromatin Imunnoprecipitation sequencing and electrophoresis mobility shift assays identified Rv1816 and Rv3249c as repressors of *mmpL3*, as well as all other *mmpS/mmpL* genes, with the exception of *mmpL6*.^154,155^ The observation that so many *mmpS/mmpL* genes share transcriptional regulators with *mmpL3* suggests that a coordinated regulatory pathway is needed for Mtb to adapt to new environments. In addition, Rv1816 also regulates *kasA* of the FAS-II pathway.^154^ This finding suggests that *mmpL3* regulation is co-coordinated with MA synthesis, despite no direct protein-protein interaction.^110^ This finding is consistent with the observation that FAS-I/FAS-II genes are downregulated following *mmpL3* knockdown or MmpL3 inhibition (Fig. 1).^22,138,156^

Repression by Rv1816 and Rv3249c is relieved upon binding to either palmitic acid or isopropyl laurate.^154^ The identification of palmitic acid as an inducer was serendipitous,^154^ but is consistent with the model that links MA synthesis with transport regulation as Mtb Fas is biased to stearic acid (C_18_).^40,41^ One model that may link Fas with MmpL3 is that as Fas begins to generate short-chain FAs, *mmpL3,* as well as other genes regulated by Rv1816/Rv3249c, is induced to ready the cell for replication. However, additional experiments would be needed to test this model.

In addition to transcriptional regulation, MmpL3 activity is post-translationally phosphoregulated by PknA, PknB, and PstP at the C-terminal domain.^126,127^ In two separate studies, Zeng J. and Adams O. demonstrated that overexpression of PknB led to increased phosphorylation of MmpL3_Msm_ at T920 and T984,^126^ while depletion of *pknA* resulted in decreased phosphorylation of the MmpL3_Mtb_ residues T893 and T910.^127^ Consistent with a repressive model, overexpression of PknB is lethal and mirrored MmpL3 perturbation lipid profiles resulting in increased TMM as MmpL3 transport was lost and decreased TDM due to lower TMM substrate.^126^ In accordance with this model, both genetic depletion of *pknA* and *pknB* and small molecule inhibition of PknA and PknB resulted in increased TMM, but no alteration in TDM levels.^127,157^ The lipid profiles of the PknA/B disruption studies are consistent with a model of unregulated MmpL3 activity in which TMM is synthesized through FAS-II upregulation and TMM export at increased rates, but without TDM conversion due to lower Ag85B expression following PknA/B disruption.^127,157^ These studies were conducted in two mycobacterial species, Msm and Mtb,^126,127,157^ suggesting MmpL3 phosphoregulation is conserved in other mycobacteria.

The phosphorylation-based inhibition of MmpL3 activity is relieved by the serine/threonine phosphatase PstP.^126^ Repression of *pstP* resulted in the loss of viability and a decrease in TMM abundance consistent with the loss of MmpL3 activity through phosphorylation.^126^ That phosphorylation of the MmpL3 C-terminal inhibits protein activity is consistent with observations that the C-terminal domain is not essential for MmpL3 activity or cell viability.^89^ Regulation by PknAB/PstP is not limited to MmpL3, and includes other lipid/MA synthesis enzymes, including Fas, FabG, HadA, AccD5, FadD32, AccA3, and Pks13,^126^ as well as Wag31,^148,149^ further linking MmpL3 activity with cell wall synthesis and division. PknAB/PstP also regulates PG synthesis,^158^ linking MA transport regulation with PG biosynthesis.

Interestingly, while depletion of *pknA* led to decreased phosphorylation of MmpL3, depletion of *pknB* did not.^127^ This may suggest differential regulatory roles of PknA and PknB, despite *pknB* being encoded immediately downstream of *pknA* in the same operon.^127^ Although PknB localizes to the dividing pole during cell division, PknB was not identified as a not identified as a part of the MmpL3 interactome,^93,110^ suggesting a transient association with MmpL3. Fascinatingly, depletion of both *pknA* and *pknB* in a double knockdown strain led to increased phosphorylation of MmpL3_Mtb_ at residues S823, T840, S868, T872, T893, and T910.^127^ As only Ser/Thr residues were phosphorylated in the *pknA/B* double knockdown strain, this suggests that other Ser/Thr kinases are involved in MmpL3 phosphoregulation. Mtb has 11 Ser/Thr kinases,^113^ including PknA and PknB, suggesting that the other 9 Ser/Thr kinases may play a role in phosphoregulating MmpL3. A study by Prisic et al identified Thr residues as the preferred phosphorylation sites for PknA, PknB, PknD, PknE, PknF, and PknH.^160^ This would suggest that the Ser residues identified to be phosphorylated by Zeng et al in the *pknA/B* double knockdown strain may be phosphorylated by PknG, PknI, PknJ, PknK, or PknL.

Additional studies are required to understand the transcriptional and post-translational regulation of MmpL3. While MmpL3 is post-translationally regulated through phosphorylation of the C-terminal domain,^126,127^ the residues identified are not conserved in all mycobacteria. These differences may result in differential post-translation regulation of MmpL3 between species. In addition, how phosphorylation of the C-terminal domain results in decreased MmpL3 activity is not clear, but may result in (i) dissociation of predicted MmpL3 homotrimers,^89,110^ (ii) dissociation of MmpL3 from other interacting proteins such as TmaT^110^ and TtfA,^93^ or (iii) delocalization from the dividing pole.

## The Therapeutic Potential of MmpL3

MmpL3 is conserved in mycobacteria as well as the closely related *Corynebacterium* spp. (CmpL1).^89,136^ MmpL3 was initially demonstrated to be essential in two studies by Lamichhane et al in a transposon screen,^161^ and by Domenech et al, who could not generate an *mmpL3* knockout.^143^ Since then, several additional lines of genetic evidence have validated this finding, including the observation that mycobacteria rapidly lose viability upon *mmpL3* knockdown or MmpL3 protein depletion.^137,138,140^ In addition, saturating transposon mutagenesis studies have failed to identify null mutants in Mtb, *Mycobacterium bovis* Bacillus Calmette-Guérin (BCG), or *Mycobacterium paratuberculosis*.^81–85,135^ However, recently, Xiong et al reported an *mmpL3* knockout in *M. neoaurum* (ATCC 25795).^139^ The method used to create this knockout strain failed to generate knockouts in Mtb^143^ and *M. smegmatis*.^29^ Therefore, why Xiong et al were able to knockout *mmpL3* in *M. neoaurum* is unclear. Some possibilities include (i) the presence of an additional (ac)TMM transporter or (ii) a lowered dependency on TMM for MM anchoring in *M. neoaurum*. Precedent for either scenario exists for closely related *Corynebacterium* species, which are viable without the TMM equivalent trehalose corynemycolate (TMCM).^90,136^ To date, the effects of MmpL3 inhibitors have not been tested in *M. neoaurum,* which may give some insights into the essentiality of MmpL3 and TMM transport in this mycobacterial species. However, in most species of mycobacteria, including clinically relevant Mtb, MAC, and MAB, *mmpL3* remains classified as an essential gene in replicating cells. This was recently highlighted in a whole genome CRISPRi knockdown screen by Bosch et al, which identified *mmpL3* as one of the most vulnerable genes in Mtb to genetic perturbation.^162^

The essentiality of MmpL3 *in vitro* translates to both *ex vivo* and *in vivo* infection models. Genetic knockdown and protein depletion models have demonstrated that Mtb rapidly loses cell viability in both infected macrophages^138,163^ and mice (C57Bl/6)^137^ following MmpL3 depletion. These observations make MmpL3 an attractive target for TB chemotherapy and efforts to identify MmpL3 inhibitors have been remarkably successful through both untargeted^20–35,37^ and targeted^25,36^ screening approaches. To date, at least 30 parental chemical scaffolds have been proposed as MmpL3 inhibitors.^20–38^

Treatment of Mtb and other mycobacteria with these inhibitors results in bactericidal effects *in vitro*,^20–38^ as well as growth inhibitory effects in *ex vivo* models.^21,22,25,26,35,164–166^ Follow-up structure–activity relationship (SAR) studies have resulted in the development of hundreds of active analogs,^22,31,35,37,165–168^ with some demonstrating activity against Mtb and MAB *in vivo*.^27,28,35,153,164,167–174^ However, it should be noted to what extent these parental compounds and their active analogs have been investigated to directly target MmpL3 varies. In addition, in many cases, SAR studies are not followed up with target validation to ensure that MmpL3 remains a target. Furthermore, several MmpL3 inhibitors have demonstrated additional mechanism of action (MOA), and few studies have been conducted to test for such off-target effects in most proposed MmpL3 inhibitors.

Adding to the success of MmpL3 inhibitors in preclinical models, SQ109 has had success in clinical trials^175,176^ and recently completed a Phase IIb clinical trial.^177^ In humans, SQ109 is well tolerated^175–177^ and leads to decreases in viable Mtb in sputum samples when taken in combination with RIF over 14 days.^176^ While SQ109 is active against MDR-TB in patients,^177^ SQ109 has a short half-life due to host drug metabolism,^178,179^ which is exacerbated when taken in combination with RIF.^175^ However, the success of SQ109 thus far validates MmpL3 as a clinical target for Mtb therapy. Moving forward, clinical trials involving other MmpL3 inhibitors with altered pharmacokinetic profiles may find additional success.

The success of SQ109 as an MmpL3 inhibitor in human TB patients shows promise for other MmpL3 inhibitors being studied and developed elsewhere. However, MmpL3 as a target is not without its limitations. Despite being a commonly identified target for inhibitors active against Mtb, these same inhibitors are not active in all mycobacterial species. Using a wide selection of previously described MmpL3 inhibitors, Li et al demonstrated that some MmpL3 inhibitors, including SQ109, have low activity against MAB and MAC species.^180^ The low activity in NTM species was scaffold specific, and some MmpL3 inhibitors such as the indolecarboxamides NITD-304 and NITD-349 demonstrate high activity against NTMs.^180^

Another limitation of MmpL3 as a target is the nonessential nature of MmpL3 in nonreplicating mycobacteria.^89,137^ In the granuloma, the growth rate of Mtb exists along a spectrum of actively replicating to nonreplicating due to factors including nutrient starvation, host derived stresses, and hypoxia.^181^ Using an inducible MmpL3 depletion strain, Li et al demonstrated that during states of nonreplication, depletion of MmpL3 did not significantly reduce the viability of Mtb.^137^ This observation is consistent with observations that the treatment of nonreplicating Mtb with indocarboxamides, AU1235, and HC2091 did not lead to significant losses in viability^22,152^; however, others do kill nonreplicating bacteria (discussed further below). Taken together, these observations indicate that strategies must be developed to overcome therapeutic limitations of targeting MmpL3.

While MmpL3, as a target, has limitations for nonreplicating mycobacteria, similar “lack of activity” observations for nonreplicating bacteria have been made for the first-line drug INH,^22,152,182–185^ which has been used clinically for nearly 70 years.^186^ In addition some MmpL3 inhibitors, including SQ109, BM212, C215, TBL-140, E11, HC2032, HC2134, HC2138, HC2149, HC2178, and HC2184, have additional effects, including PMF uncoupling,^25,31,152,165^ and some have been demonstrated to be active against nonreplicating persistent Mtb.^22,31,152,165^ While these off-target effects do not add to the therapeutic potential of MmpL3, they do suggest that limitations in MmpL3 inhibition can be overcome through secondary MOA. In addition, mycobacteria are treated using drug combinations, and inclusion of drugs active against nonreplicating mycobacteria such as Bedaquiline,^187^ RIF, and others^188^ could overcome therapeutic limitations of MmpL3. MmpL3 inhibitors have also demonstrated high activity against clinical monodrug-resistant, MDR, and XDR-Mtb strains.^26,27,29,32,35,165,171^

Furthermore, there have been no report of resistant mutants isolated from patients who received SQ109 during clinical trials. However, analysis of >45,000 whole genome sequences from clinical samples biased toward drug-resistant strains identified nonsynonymous mutations, although at low frequencies.^128^ These mutations included ones known to cause resistance to preclinical MmpL3 inhibitors, including a V210A mutant that has been demonstrated to be resistant to SQ109 *in vitro*.^34^ While follow-up studies to this finding have not been conducted to determine the level of resistance such strains have against SQ109 and other MmpL3 inhibitors being developed, these findings do emphasize the need for active surveillance of clinical strains resistant to MmpL3.

One possible silver lining, although, is the observation that *mmpL3* mutants resistant to MmpL3 inhibitors are hypersusceptible to RIF,^25,189^ suggesting a co-therapy of RIF and an MmpL3 inhibitor could reduce the frequency of resistance (FoR) to MmpL3 inhibitors in the clinic. Taken together, while MmpL3 does have limitations as a target, strategies are available to overcome them and MmpL3 remains a viable therapeutic target.

## How are Putative MmpL3 Inhibitors Identified and Validated?

MmpL3 remains a common target, owing to its continued identification as the target of a broad number of compounds with varying chemical scaffolds (Fig. 5a–f). However, most MmpL3 inhibitors have been identified through two methods; (i) the isolation and whole genome sequencing of *mmpL3* mutants resistant to an inhibitor^20–37,164–169,171,172,189,190^ and (ii) the observation that treatment of mycobacteria with proposed MmpL3 inhibitors leads to the accumulation of TMM and a decrease in TDM in whole cell lipid extracts.^21,22,25,27,29,31–33,35,164,166^

While these two methods have served as early indicators for the MOA of these compounds, they are confounded by the additional observations that (i) MmpL3 inhibitors can have multiple MOA and can kill Mtb in nonreplicating states^31,152,165^ and (ii) disruption of the PMF can lead to similar lipid abundance profiles as cells treated with proposed MmpL3 inhibitors.^152^ These two observations previously brought into question the true target of proposed MmpL3 inhibitors.

**FIG. 3.**
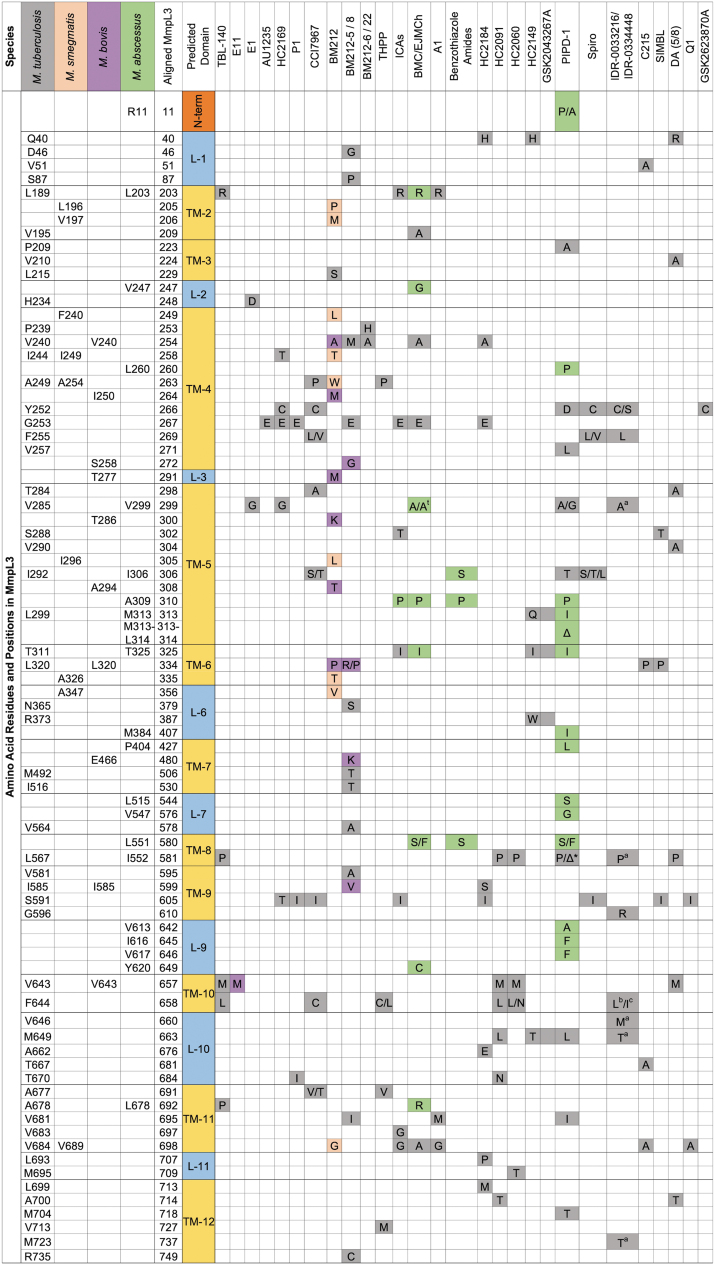
Amino acid substitution localization for MmpL3 inhibitor-resistant mutants. A matrix that demonstrates the amino acid position and substitution for nonsynonymous mutations found in MmpL3 inhibitor-resistant mutants. The matrix includes inhibitors for which resistant mutants have been identified in four species, including *Mycobacterium tuberculosis* (*gray*), *Mycobacterium smegmatis* (*orange*), *Mycobacterium bovis* BCG (*purple*), and *Mycobacterium abscessus* (*green*). MmpL3 protein sequences were aligned and indicate orthologous positions between species. *Substitutions in position 581 of the aligned protein for PIPD-1 were discovered in either the *M. tuberculosis* (P) or *M. abscessus* (Δ), A^†^ indicates substitution in *M. tuberculosis* background at the 299 position of the aligned sequence (*M. tuberculosis—*V285). ^a^Indicates secondary substitutions made in the *M. tuberculosis* F255L background isolated from IDR-0033216, ^b,c^Indicate tertiary substitutions sequenced from AU1235 resistant mutants in *M. tuberculosis* F255L/L567P and F255L/V646M backgrounds, respectively. BCG, Bacillus Calmette-Guérin; L, loop; TM, transmembrane.

**FIG. 4.**
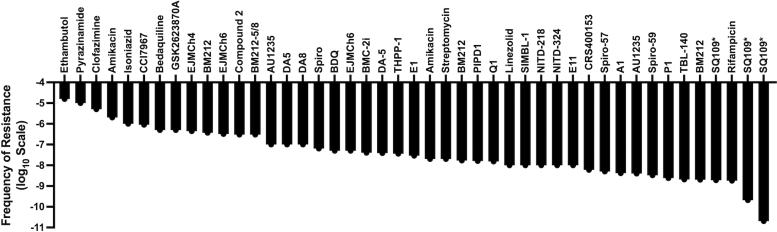
Mycobacteria have a moderate FoR to MmpL3 inhibitors. FoR plot for MmpL3 inhibitors as well as other TB drugs against mycobacteria.^21,23,26–31,33–36,164,165,167,169,193,205–213^ *Indicates FoR for SQ109 measured in *Helicobacter pylori*. FoR, frequency of resistance; TB, tuberculosis.

The isolation and whole genome sequencing of mutants resistant to novel inhibitors can act as an early indicator of the cellular target. Mutants resistant to MmpL3 inhibitors have been isolated in multiple species, including Mtb, *M. smegmatis*, *M. bovis* (BCG), and *M. abscessus* (Fig. 3).^20–37,164–169,171,172,189,190^ The FoR to MmpL3 inhibitors generally ranges from 10^−7^ to 10^−9^ (Fig. 4),^20–37,164–169,171,172,189,190^ but can vary between species.^30^ Further, mutations to MmpL3 inhibitors primarily occur in regions encoding TMD surrounding the central vestibule (Fig. 3)^121^ where inhibitors bind to MmpL3.^121,122^

While the isolation of *mmpL3* mutants against proposed MmpL3 inhibitors has a good track record as an early indicator that a compound targets MmpL3, it is neither proof that MmpL3 is either the target nor the only target of an inhibitor. For example, resistant mutants isolated against THPP-based MmpL3 inhibitors by both Ioerger as well as Remuinan and their respective colleagues indicated that THPP inhibits MmpL3.^24,35^ However, a protein pull-down study conducted by Cox et al using chiral enantiomers of THPP, GSK729 (active), and GSK730 (inactive) identified EchA6, but not MmpL3, as a strong binder of THPP (GSK729).^191^

Targeted mutagenesis of *echA6* conferred resistance to THPP both *in vitro* and *in vivo* (murine), indicating that EchA6 was, at least, an additional target of THPP.^191^ These observations led Cox et al to hypothesize that MmpL3 acted as a drug importer, and that mutations in *mmpL3* blocked this function.^191^ However, *mmpL3* mutants resistant to AU1235 and BM212 demonstrated no difference in cellular accumulation of these inhibitors, indicating MmpL3 is not an importer for these inhibitors.^29,30^ Later studies would also validate MmpL3 as a target of THPP based on protein–inhibitor interaction studies, including biolayer interferometry (BLI) and SPR.^118^ In addition to THPP, SQ109 also has multiple proposed targets, including MmpL3,^34^ PMF uncoupling,^152^ and menaquinone biosynthesis by targeting MenA.^192^ As an additional limitation to this primary method, no Mtb resistant mutant has been identified against SQ109 either *in vitro* or reported from clinical trials. FoR studies in *Helicobacter pylori* have indicated that the FoR for SQ109 is 10^−9^ to 10^−11^ (Fig. 4), which is likely due to the multitarget nature of SQ109.^193^ Due to this limitation, resistance studies for SQ109 have utilized *mmpL3* mutants isolated against other inhibitors that are cross-resistant to SQ109.^34^ However, taken together, observations for THPP and SQ109 demonstrate how isolation of *mmpL3* mutants is not enough to validate MmpL3 as a target, nor does it rule out the possibility of additional MOAs.

The most common method used to validate MmpL3 as the target following *mmpL3* mutant sequencing is to perform lipid profiling of mycobacteria treated with an MmpL3 inhibitor. This method relies on the comparison of relative lipid abundance of TMM and TDM in MmpL3 inhibitor treated versus untreated control cells. Following MmpL3 disruption, mycobacteria accumulate TMM as it can no longer be transported across the CM.^29,117^ This leads to a decrease in TDM due to a lack of TMM substrate in the MM. These lipid profiles are consistent between mycobacteria treated with MmpL3 inhibitors^152^ and inducible MmpL3 depletion strains.^89,118,137^

However, MmpL3 is powered by proton translocation and PMF uncoupling can lead to MmpL3 perturbation. Studies by Li et al generated similar lipid profiles from *M. smegmatis* cells treated with MmpL3 inhibitors or PMF uncouplers such as CCCP and nigericin.^152^ Further, studies conducted by multiple groups have indicated that MmpL3 inhibitors such as SQ109, BM212, C215, TBL-140, E11, HC2032, HC2134, HC2138, HC2149, HC2178, HC2184, and SIMBL-2 can disrupt one or more components of the PMF.^25,31,33,117,152,165^ These observations brought into question whether proposed inhibitors inhibit MmpL3 through direct protein binding or indirectly through PMF uncoupling.

More recently, several protein binding assays have indicated that MmpL3 inhibitors bind to MmpL3 directly,^118,121,122^ and that PMF uncoupling is likely a secondary effect either independent of MmpL3 inhibition or as a result of conformational changes following binding (discussed further below).^122^ Taken together, while the isolation of *mmpL3* mutants coupled with lipid profiling can act as an early indicator that a compound is an MmpL3 inhibitor, additional studies are required to validate MmpL3 as the primary target. Some methods that have been used to test for target specificity (discussed further below) include transcriptional profiling,^22,138,156^ sensitivity testing in *mmpL3* knockdown strains,^36^ fluorophore displacement,^118^ and metabolic profiling.^23^

Due to the limitations of both genomic sequencing and lipid profiling, efforts were made to create assays that more directly measured protein–inhibitor interactions. The aforementioned spheroplast assay was one such assay,^117^ which shows that MmpL3 inhibitors prevent (ac)TMM flipping across the CM. However, this assay is not without its limitations. The generation of spheroplasts can be difficult as mycobacteria lacking their cell wall are vulnerable to spontaneous lysis, as mentioned by the developers of the assay.^117^ In addition, the confounding finding that SQ109 did not inhibit TMM flipping is still unclear, as more direct biochemical assays have demonstrated that SQ109 does interact with MmpL3 in a manner similar to the other inhibitors (discussed shortly).^118,121^

A separate assay developed by Li et al uses a competitive binding of MmpL3 with fluorescently labeled analogs of known MmpL3 inhibitors.^118^ Briefly, fluorescently tagged MmpL3, either in cells or as purified protein, is incubated in the presence of fluorescently labeled MmpL3 inhibitors called North probes; following incubation, MmpL3 is challenged with unlabeled MmpL3 inhibitors, which competitively bind and displace the North probes. Measuring the loss of probe fluorescence following inhibitor exposure allows for the determination of MmpL3-inhibitor interaction. This method has several advantages to the spheroplast assay. First, it is amenable to both biochemical and live cell assays.^118^ Second, the competitive binding assay is insensitive to PMF uncoupling, whereas the spheroplast assay is not.^117,118^ However, this binding assay is not without limits and could be susceptible to false positives through compounds that dislodge the North probes through nonspecific interactions with MmpL3 rather than competitive binding.

Finally, progress has been made in purifying MmpL3 form both Mtb and *M. smegmatis*. This has allowed for more direct protein inhibitor interactions to be generated by SPR and BLI, which allow kinetic measurements to be made.^118^ However, this assay has been limited by solubility issues for some proposed MmpL3 inhibitors such as BM212, which could not used in either SPR or BLI.^118^ Nonkinetic structural assays have also been conducted through X-ray crystallography. These studies have shown that several proposed MmpL3 inhibitors, including SQ109, AU1235, Rimonabant, ICA38, Spiro, and NITD-349, directly bind to MmpL3 in a similar manner, despite their broad differences in structure.^121,122^

Taken together, researchers now have the tools to test directly whether or not a proposed MmpL3 inhibitor directly binds to and inhibits MmpL3 both biochemically and in live bacteria. However, these assays do not preclude the possibility of secondary and off-target effects. We have compiled the summary of proposed MmpL3 inhibitors identified to date, not including the expansive analogs generated by SAR studies, and what assays have provided evidence that each of these compounds targets MmpL3 (Supplementary Table S1).

## Impacts of MmpL3 Disruption

Because MmpL3 is involved in MM synthesis, it would be expected that perturbation of MmpL3 would put mycobacteria into a state of cell wall stress. Mycobacterial reporter strains for cell wall stress were generated through -*gfp* and -*lacZ* fusions to *iniB*, which, along with downstream genes *iniAC*, are highly upregulated following INH and EMB treatment.^194,195^ These reporter systems are largely insensitive to non-cell wall inhibitors^194,195^ and were used in screens that identified the MmpL3 inhibitors DA-5, DA-8, and E11.^31,34^ These observations are consistent with transcriptional profiles of Mtb treated with SQ109, HC2091, and following *mmpL3* knockdown, which resulted in increased *iniBAC* expression.^22,138,156^ These findings support a cell wall stress model for MmpL3 inhibitor treatment even in the case of E11, which has the added effect of PMF disruption.^31^

As described earlier, the inhibition of MmpL3 leads to a decrease in TMM transport resulting in TMM accumulation in the inner leaflet of the CM. This was most clearly demonstrated by Xu et al using *M. smegmatis* spheroplasts and dual TMM fluorescent metabolic probes and TMM degradation assays.^117^ They demonstrated that treatment of *M. smegmatis* spheroplasts with MmpL3 inhibitors BM212 and AU1235 resulted in decreased TMM flipping to the outer leaflet of the CM.

Consistent with alterations in the makeup of the cell wall, it was reported that MmpL3 inhibition leads to increased cell hydrophobicity in *M. smegmatis* treated with AU1235^189^ and cell permeability following *mmpL3* silencing in Mtb.^36^ Drug combination studies have demonstrated that MmpL3 disruption leads to RIF hypersusceptibility^25,36,103,196^; additionally, MmpL3 perturbation leads to increased susceptibility to PG synthesis inhibitors.^36,196^ Scanning electron and transmission electron micrographs have also shown alterations to cell morphology following MmpL3 inhibition^34,153^ which is consistent with cell wall alterations. RIF susceptibility is tied to cell permeability^197^ and the observation that MmpL3 inhibition leads to PG dysfunction suggests association between MA biosynthesis and PG synthesis as discussed above.

Treatment of bacteria can lead to whole cell changes and alter transcriptional and metabolic profiles. Early studies into the MOA of SQ109 included a large transcriptional profiling study by Boshoff et al, who conducted over 400 microarray experiments of Mtb cultured and treated under different conditions.^156^ The resulting profiles placed SQ109 (then diamine 109) with the AG biosynthesis inhibitor EMB. However, the gene expression profiles of SQ109 included the downregulation of genes involved in MA biosynthesis, including *fas, fadA2, pks16,* and the *fabD-acpM-kasA-kasB-accD6* operon (Fig. 1, red highlight). While these genes were downregulated in the SQ109 profiles, they were upregulated in the EMB and INH profiles, suggesting SQ109 had a MOA unique from other cell wall inhibtiros.^156^ Later, similar patterns for downregulated genes were observed in our laboratory from RNAseq profiles of Mtb treated with SQ109 or HC2091.^22^

Similarly, the RNAseq profile from an Mtb *mmpL3* knockdown strain also identified the downregulation of MA biosynthesis genes.^138^ The profiles generated in these three studies were highly similar and generated in the presence of SQ109, which decreases the Δψ,^152^ HC2091, which does not affect the Δψ,^22^ and following *mmpL3* depletion, which, presumably, leads to an increased Δψ, as observed by Li et al.^118^ This suggests that the transcriptional responses identified were highly specific to MmpL3 disruption and independent of secondary membrane energetic effects.

The consistent differential profiles generated by MmpL3 disruption not shared by other cell wall inhibitors suggest that mycobacteria can sense when MmpL3 specifically is inhibited. As a consequence of these expression changes, the associated metabolites should also decrease in abundance. Consistent with this model, metabolomic profiles generated by Zampieri et al of *M. smegmatis* treated with GSK2623870A identified a decrease in trehalose 6-phosphate,^23^ the activated form of trehalose that serves as a substrate to make TMM.^74^ In addition, Zampieri et al identified that FAS proteins, such as Fas, were primarily affected in a proteomic analysis.^23^

Of the three profiles generated to date, the *mmpL3* knockdown profile was the most robust, with several regulatory pathways identified as differentially expressed, in addition to MA biosynthesis.^138^ The regulatory pathways may be involved in the sensing of changes to the cell envelope following MmpL3 perturbation. However, it is also possible that this profile includes genes responding to the increase in the Δψ following *mmpL3* knockdown.^118^ Based on the transcriptional profiles, we know that Mtb represses genes involved in both the FAS-I and FAS-II pathways. Mtb also upregulates expression of the cell wall stress operon *iniBAC* in response to MmpL3 disruption.^22,138,156^

How Mtb specifically senses MmpL3 disruption, and differentially regulates its genes from other cell wall inhibitors such as EMB and INH, is not clear. One model may suggest that Mtb senses changes in CM fluidity following TMM accumulation or TMM accumulation in the inner leafelt.^117^ Such changes would not occur in INH-treated cells, as FAS-II disruption from INH would not directly affect CM fluidity as CM lipids are generated through FAS-I.^198^ If so, then this may be through either the alternative sigma factor, SigE (Rv1221), or the two-component system regulator MprAB (Rv0981 and Rv0982), which responds to cell wall stress^199,200^ and whose regulons were upregulated in the *mmpL3* knockdown profile.^138^

Of note, the transcriptional effects following MmpL3 disruption are primarily repressive, and few genes are observed to be upregulated in response to MmpL3 inhibition.^22,138,156^ One notable change was the induction of osmotic stress genes, *oprA* (*Rv0516c*) in the *mmpL3* knockdown profile,^138^ suggesting that the bacteria are experiencing osmotic stress. Other genes upregulated in these profiles include SigE and MprAB regulated genes in the *mmpL3* knockdown profile, and the *iniBAC* cell wall stress signature genes in all three profiles.^22,138,156^ However, these transcriptional signatures are not specific to MmpL3 disruption and are upregulated in Mtb in other stresses.^156^

Several reporter strains were built around genes that are upregulated following specific stresses.^194,195^ However, limited genes that are upregulated in response to MmpL3 disruption in Mtb does not lend itself for the use of building a reporter strain. While the gene repression signature following MmpL3 disruption is unique compared to FAS-II or AG synthesis inhibition,^156^ disentangling this gene repression signature from cell death or transcriptional repression from RNA polymerase and DNA gyrase inhibitors is difficult.

Whatever differences may exist in the exact nature of each inhibitor's MOA, it remains clear that MmpL3 inhibition leads to the accumulation of TMM and a decrease in TDM. As discussed above, TMM covalently anchors the MM to the rest of the cell wall forming mAGP, and TDM, the metabolic product of Ag85 and TMM, acts as a major penetration barrier in mycobacteria.^97^ Mycobacteria treated with MmpL3 inhibitors have increased cell permeability similar to strains with lower MmpL3 function leading to increased antibiotic efficacy.^36,197^

## MmpL3 Protein–Inhibitor Interactions

Despite the limitations of the two primary methods used to identify MmpL3 inhibitors listed in the previous section, a competitive binding assay developed by Li et al has demonstrated direct interaction for many MmpL3 inhibitors with MmpL3 (discussed in a previous section).^118^ Leveraging the displacement property, these North probes can be used to determine direct MmpL3 interaction in live cell mycobacteria through competitive binding using flow cytometry by measuring the relative fluorescent intensity. This system has already been used in three separate studies to demonstrate direct interaction of MmpL3 with inhibitors SQ109, NITD-304, NITD-349, BM212, AU1235, THPP1, HC2032, HC2060, HC2091, HC2099, HC2134, HC2138, HC2149, HC2169, HC2178, HC2184, C215, SIMBL-1, and SIMBL-2.^25,33,118^ Further, the results of SQ109, NITD-304, NITD-349, AU1235, and THPP1 have were backed by protein binding data, including BLI and SPR.^118^

However, while this competitive binding assay does allow for the simple measurement of direct interaction between MmpL3 and an inhibitor in live cells, this competitive binding assay does not rule out the possibility of additional effects. Inhibitors like SQ109, BM212, C215, TBL-140, and E11, and many of the listed HC2 compounds have the additional property of PMF disruption.^25,31,152,165^ Compounds like BM212 and SQ109 were both demonstrated bactericidal properties against Mtb in nonreplicating states^152^ when MmpL3 is dispensable for viability.^137^ In addition, evidence does support secondary protein targets for both THPP^191^ and SQ109.^192^

**FIG. 5.**
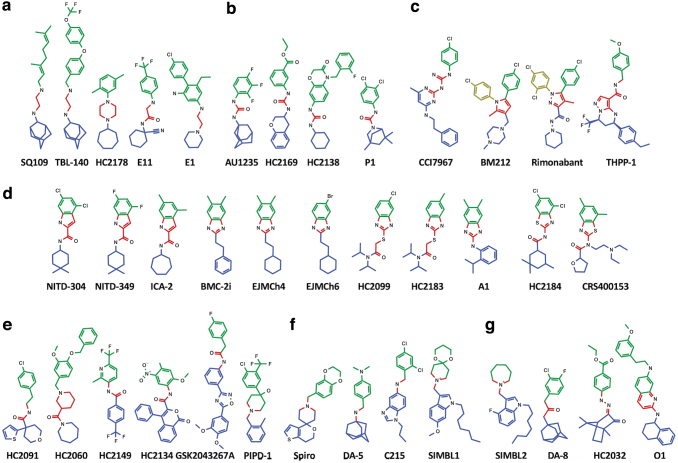
MmpL3 inhibitors share distinguishing and overlapping features. MmpL3 inhibitors fall into seven distinct classes of inhibitors based on shared Central core chemical groups, including diamines/acetamides **(a)**, ureas/guanidines **(b)**, pyrole/pyrazoles **(c)**, indoles/imidazoles/thiazoles **(d)**, amides **(e)**, amines **(f)**, and a seventh class of unshared core chemical groups **(g)**. *Colored structures* indicate shared chemical groups found between all MmpL3 inhibitors, including the Central nucleophilic/basic core group (*red*), as well as the lipophilic/hydrophobic Northern (*green*) and Southern (*blue*) groups. Noted exceptions are the additional North-Western chemical groups (*yellow*) found in BM212 and Rimonabant. North, Central, and South chemical nomenclature is adopted from framework proposed by Guardia et al.^170^

While the specific chemical structure varies between proposed MmpL3 inhibitors, the compounds can be broadly classified into seven primary categories based on shared core structures (Fig. 5a–g). The seven classes consist of diamine/acetamides, ureas/guanidines, pyrole/pyrazoles, benz-amides/indoles/imidazole/thiazoles, amides, amines, and a seventh class of scaffolds that do not share a common core structure (Fig. 5a–g).

For simplicity of description, we adopt nomenclature from Guardia et al^170^ and described the shared core structures as the Central chemical groups (Fig. 5a–e, red). The Central cores are typically composed of nucleophilic/basic residues, and even in the seventh chemical class, which lack a shared core structure, the inhibitors share a Central core with similar chemical properties (Fig. 5g). Again, adopting nomenclature from Guardia et al, the Central cores are flanked by lipophilic/hydrophobic Northern (Fig. 5a–g Green) and Southern (Fig. 5a–g Blue) groups. The Northern group is typically in the form of a substituted aryl group, such as benzene, while the Southern group typically consists of cyclic alkyl groups.

While the shared chemical cores exist for nearly all MmpL3 inhibitors, additional groups have been identified for BM212 and Rimonabant, each of which has additional substituted benzene rings classified here as North-Western groups (Fig. 5c, Yellow). A review of the modeled and co-crystal structures indicates that the chemical domains align to specific MmpL3 binding subdomains (SD-1 to SD-5) identified by Zhang et al (Fig. 2b).^121^ The Northern groups are typically found in the SD-3 toward the periplasmic side of the Asp-Tyr residues.^121^ Exceptions to this are noted for BM212 and Rimonabant, where the Northern groups bind to SD-2 and the North-Western groups bind to SD-1.^121^ The nucleophilic/basic Central groups interact with the essential Tyr-Asp groups in SD-4 (Fig. 2c).

The Central groups typically bind to the Tyr residues through H-bonding or noncovalent interactions.^121^ This binding disrupts Asp-Tyr pairing, preventing H^+^ translocation that powers MmpL3 function.^121^ The Southern group of MmpL3 inhibitors is typically located in SD-5 and acts as stabilizers through hydrophobic interactions.^121^ Based on these binding patterns, while specific protein–inhibitor interactions exist, as exemplified through different inhibitory concentrations for structurally similar series identified in SAR studies,^22,31,37,165–168,170,201,202^ MmpL3-inhibitor interactions are characterized by a limited number of general protein inhibitor binding motifs.

## *mmpL3* Drug-Resistant Mutations May Affect MmpL3 Structure Function and Mycobacterial Growth

Forward genetic screening for *mmpL3* mutants resistant to MmpL3 inhibitors has been one of the primary methods used to identify MmpL3 inhibitors. Mutations in *mmpL3* are primarily located in codons encoding residues located in the central vestibule.^20–37,164–169,171,172,189,190^ Some researchers have noted that these mutant strains have *in vitro* growth defects.^25,118,189^ Protein gels of trypsin-digested wild-type (WT) and mutant MmpL3 demonstrated altered folding motifs in MmpL3^118^ and modeled substitutions in the MmpL3 protein structure suggests that substitutions lead to changes in the protein folding around the central vestibule.^121,128^ A recent study by McNeil et al demonstrated that *mmpL3* mutants isolated against MmpL3 inhibitors have altered membrane potential (Δψ).^189^ The altered Δψ had global effects on *M. smegmatis* cells, including lowered efflux, increased cell permeability, RIF hypersusceptibility, and altered TMM/TDM profiles similar to MmpL3 inhibition.

These altered physiologies suggested that mutations in *mmpL3* lowered MmpL3 function resulting in lower growth rates.^189^ Additional studies by McNeil et al demonstrated that passaging these mutants in the presence or absence of an MmpL3 inhibitor led to the selection of compensatory mutations in the form of secondary, as well as tertiary, mutations in *mmpL3*.^189,190^ These additional substitutions often arose in the central vestibule in TMD adjacent to the primary substitution.^189^ The passaged mutants demonstrated a reversion to WT levels of growth, suggesting a return to normal protein function, while maintaining inhibitor resistance.^189,190^ While this allowed for insights into the biology and structure-function relationship of MmpL3, it also supports the potential selection of *mmpL3* mutants resistant to MmpL3 inhibitors in the clinic.

Passaging the mutants in the presence of everincreasing MmpL3 inhibitor concentrations only led to additional mutations and increased resistance without a growth defect.^190^ The isolated tertiary *mmpL3* mutants were also cross-resistant between IDR-0033216, IDR-0334448, AU1235, and SQ109.^190^ Based on this observation, it is possible that swapping between MmpL3 inhibitors in the clinic may select for additional mutations and higher resistance. The compensatory mutants also did not have increased susceptibility to RIF like primary *mmpL3* mutants.^190^ How these findings translate to the clinic will only be answered with time, but underscore the need for vigilant antibiotic resistance monitoring.

## Screening for MmpL3 Inhibitors

MmpL3 inhibitors continue to be the most diverse class of inhibitors that share a single target for Mtb. Because of the slow growth of Mtb and *M. bovis*, untargeted screening approaches for MmpL3 inhibitors through the isolation and sequencing of resistant mutants are largely inefficient and time-consuming. An alternative approach of using the rapidly growing mycobacteria *M. smegmatis* or *M. abscessus* is limited by observations that not all MmpL3 inhibitors are active in these two species.^25,180^

Because MmpL3 is such a common target with high therapeutic potential, screening platforms to identify these inhibitors are highly sought. While reporter strains such as the *iniB* reporter system have identified some MmpL3 inhibitors,^31,34^ the low specificity for MmpL3 limits the use of this reporter system to its original intended purpose of identifying cell wall inhibitors. In addition, while reporter systems have been built around genes highly expressed following target inhibition, MmpL3 inhibition largely leads to the downregulation of signature genes^156^ limiting the ability to develop a reporter around transcriptional signatures following MmpL3 disruption.

Alternatively, whole cell morphological changes were recently described to differentiate the inhibitors by their specific MOA using a system called Morphological Evaluation and Understanding of Stress (MorphEUS).^203^ While this system is unique in its ability to identify both primary and secondary MOAs, it is limited by specificity, and places inhibitors in broad stressor categories like cell wall stress. Recently, however, four potential screening methods have been proposed as screening platforms for MmpL3 inhibitors. These include (i) a high-throughput metabolic screening platform,^23^ (ii) a whole cell targeted mutant screen that uses *mmpL3* mutants,^25^ (iii) a two-way *mmpL3* regulatory phenotypic reporter system,^36^ and (iv) a competitive binding assay that uses MmpL3 binding fluorophores.^118^

The MOA of inhibitors can lead to differential responses in bacteria, which can be measured in a number of ways. One of these measurable responses are the metabolic changes bacteria undergo following the inhibiton of specific metbaolic pathways. Using a high-throughput metabolic analysis platform, Zampieri et al identified several MmpL3 inhibitors through differential metabolic profiles.^23^ They observed that 6 inhibitors from a library of 212 compounds led to repeated decreases in the metabolite α,α-trehalose-6-phosphate and neighboring lipids, precursors to TMM.

Two of the inhibitors, GSK1829729A and GSK1829728A, were reported to be chemically similar to THPP and isolation of resistant mutants to a third compound, GSK2623870A, identified *mmpL3* mutants. While not specifically designed to identify MmpL3 inhibitors, this system was fast and accurate at identifying the MOA of mycobacterial growth inhibitors. This system is not specifically limited to mycobacteria and could feasibly be applied to other bacterial and pathogenic species. Although this system can be applied to larger libraries, the technological and computational requirements to perform this assay are still somewhat limiting and require specific expertise.

The second MmpL3 inhibitor screening platform was developed in our laboratory and was built on the observation that *mmpL3* mutants have broad cross-resistance to MmpL3 inhibitors,^22,25,28,32,34,36,118,165,168,190^ but low cross-resistance to non-MmpL3 inhibitors.^25,27,28,32,34,35,37,118,164,168,189^ Another observation previously made was that the amount of cross-resistance depended on the specific combination of *mmpL3* mutant and MmpL3 inhibitor tested.^118^ To overcome potential limitations, we generated a pool of 24 unique *mmpL3* mutant isolates against 5 structurally unique MmpL3 inhibitors.^22,25^

We hypothesized that MmpL3 inhibitors would select for the mutant(s) with the highest resistance, while non-MmpL3 inhibitors would equally affect all the mutants. The resulting *Targeted Mutant Screening* assay compared the effect inhibitors have on the growth (OD_600_) of WT versus the *mmpL3* mutant pool, in which the mutants would be less effected by an MmpL3 inhibitor. Using this mutant assay, we screened a small library of 174 Mtb growth inhibitors, including previously described MmpL3 inhibitors SQ109, C215, and HC2091.^20,22,25,34^ The results of our assay identified controls SQ109 and C215, as well as HC2060, HC2091, HC149, HC2169, and HC2184, which were used to generate the *mmpL3* mutants used in the assay.^25^ In addition to these seven compounds, we also identified six other inhibitors HC2032, HC2099, HC2134, HC2138, HC2178, and HC2183.^25^

Overall, the screen was highly successful and confirmed hits induced lipid profiles consistent with MmpL3 inhibition and were positive for the competitive binding assay described above.^25,118^ However, this system has limitations. For one, we did not observe differences in the inhibitory effects for Rimonabant between the WT and *mmpL3* mutant pool.^25^ Rimonabant is highly similar to BM212, which demonstrates cross-resistance to a very small number of *mmpL3* mutants,^118^ which were not included in our pool. Future versions of this screen may seek to include *mmpL3* mutants isolated against BM212 or Rimonabant to broaden the screening potential of this system.

The third screening platform uses strains that differentially express *mmpL3.* An early version of this screen was originally proposed by Li et al, who observed inducible knockdown of *mmpL3* to subinhibitory levels rendered Mtb sensitive to MmpL3 inhibitors.^137^ While promising, this system was limited by synthetically lethal combinations with other TB drugs such as RIF.^137^ Alternatively, Zhang as well as Kozikowski, and their respective colleagues, demonstrated that *mmpL3* overexpression mycobacterial strains, expressing either WT or resistant mutant *mmpL3* alleles, conferred high resistance to MmpL3 inhibitors.^121,166^ The overexpression strain created by Zhang et al did not confer cross-resistance to INH,^121^ but further investigation into the screening potential of overexpression strains like these was not conducted.

However, recently, Grover et al demonstrated that a combined dual regulatory strain of Mtb that either repressed or overexpressed *mmpL3* was highly accurate at identifying MmpL3 inhibitors from a library of 220 Mtb growth inhibitors.^36^ The authors of this study demonstrated that *mmpL3* knockdown sensitized bacteria to MmpL3 inhibitors as previously described. However, this knockdown also made Mtb hypersensitive to non-MmpL3 inhibitors such as RIF, clarithromycin, erythromycin, fidaxomicin, and fusidic acid, as well as β-lactams.^36^ Conversely, overexpression of *mmpL3* resulted in decreased activity of MmpL3 inhibitors, but did not lead to differential inhibitory effects for non-MmpL3 inhibitors.^36^ The recognition of a bidirectional shift that only occurred for MmpL3 inhibitors indicated that this system could be used to screen MmpL3 inhibitors, which was applied to a library of 220 Mtb growth inhibitors. The results of this screen identified several previously described MmpL3 inhibitor scaffolds, including THPP-, Spiro-, Urea-, Pyrole-, PIPD-, and oxadiazole-like compounds, as well as a novel guanidine-based MmpL3 inhibitor CCI7967.^36^ The identification of CCI7967 was backed by the isolation of *mmpL3* mutants resistant to CCI7967. This screening platform overcomes the limitations of the Targeted Mutant Screening platform by identifying Pyrole-based MmpL3 inhibitors such as BM212.^25^

However, this Two-Way Regulation screen, along with the Targeted Mutant Screen, shares a common limitation of requiring compounds to be tested against both the WT reference strain as well as the experimental *mmpL3* strain(s). While these screens are not burdensome for larger pharmaceutical companies or smaller compound libraries for academic institutes, such as the ones conducted in either screen, the doubling of resources required to run these screens could become costly for larger libraries. This limitation renders both screening platforms to be used as mechanisms to identify MmpL3 inhibitors from hits from larger HTS. This limitation could be overcome in the fourth potential screening platform described next.

Finally, the fourth potential screening platform proposed uses the competitive binding assay mentioned above, using the North series of MmpL3 binding probes.^118^ This competitive binding assay utilizes MmpL3 inhibitor scaffolds such as ICAs, AU125, or the SIMBL covalently linked to the commercially available fluorophore TAMRA through click chemistry.^118^ These MmpL3-fluorophores co-localize with MmpL3 in dividing *M. smegmatis* cells and are demonstrated to interact with MmpL3 based on SPR and BLI.^118^ Using flowcytometry, researchers can measure the relative fluorescence intensity of cells treated with the North probes, which decreases in the presence of a competing MmpL3 inhibitor. This competitive binding assay is insensitive to non-MmpL3 inhibitors such as RIF and INH, as well as PMF uncouplers such as CCCP.^118^

This screen works in both whole cell mycobacteria, as well as isolated MmpL3 protein, adding diversity not available to the previously described Targeted Mutant and Two-Way Regulation screens described above. In addition, both the whole cell and biochemical assays can measure MmpL3 binding within hours, overcoming the slower times required for the other two screen platforms, which take place over days due to the slow growth of mycobacteria.^25,36^ This screen also has the potential to be used to conduct SAR campaigns directly against MmpL3 rather than whole cell bacteria. As mentioned above, SAR campaigns can result in either gain or loss in activity through the modification of parental structures. However, it has never been clear if changes in activity following structural alteration of parental compounds was due to changes in cell permeability or protein binding affinity. Utilizing *in vitro* and whole cell aspects of the competitive binding assay, it may be possible to delineate permeability from changes in binding affinity.

While this system has yet to be tested in a screen, the rapid nature of this assay coupled with the direct measurement of MmpL3 binding without the requirement for comparative strains suggests that this assay will likely make an efficient platform to screen MmpL3 inhibitors in a large library. However, this screen is limited and could identify noninhibitory MmpL3-interacting substrates that could also lead to probe displacement. Furthermore, biochemical screens have previously identified metabolic inhibitors devoid of whole cell activity due to factors such as cell impermeability.^204^ Therefore, this screening platform will still require secondary assays to demonstrate whole cell activity.

It is clear that MmpL3 inhibitors will continue to be identified through the traditional untargeted approaches of isolating *mmpL3* mutants, but with the invention of the novel screening systems listed in this study, the rate of identification will likely increase dramatically. While SQ109 has demonstrated great drug potential so far, if SQ109 were to fail in clinical trials, then it is likely that another MmpL3 inhibitor from the ones identified in the last decade could take its place.

## Concluding Remarks

MmpL3 continues to be an attractive target for TB therapy. The essential nature of the protein both *in vitro* and *in vivo* and the clinical success of SQ109 so far support further development of MmpL3 inhibitors. Protein localization and interactome studies have demonstrated that MmpL3 complexes with other cell wall synthesis metabolic pathways as part of the mycobacterial divisome. While some questions still remain, including how MmpL3 complexes with TMM in the CM, live cell microscopy and biochemical evidence demonstrate that MmpL3 plays a direct role in TMM transport across the CM.

In addition, biochemical studies have clearly demonstrated that MmpL3 directly binds to many proposed inhibitors described in the literature. These direct protein–inhibitor interactions support the isolation of resistant *mmpL3* mutants and lipid profiling as good early indicators of the MOA of identified inhibitors. Furthermore, the similar chemical properties and protein localization of these inhibitors indicate a general threshold of what makes an MmpL3 inhibitor and how they bind to MmpL3. While additional questions still remain for MmpL3 regarding regulation, protein-protein interaction, and function, the insights gained in the last decade have advanced our understanding of the role MmpL3 plays in cell biology of mycobacteria.

## Supplementary Material

Supplemental data

Supplemental data
