# Supplementary figures and images for: Molecular Mechanisms of MmpL3 Function and Inhibition
Source: Microb Drug Resist. 2023 May 4;29(5):190–212. doi: 10.1089/mdr.2021.0424 (PMC10171966; doi:10.1089/mdr.2021.0424)

a.

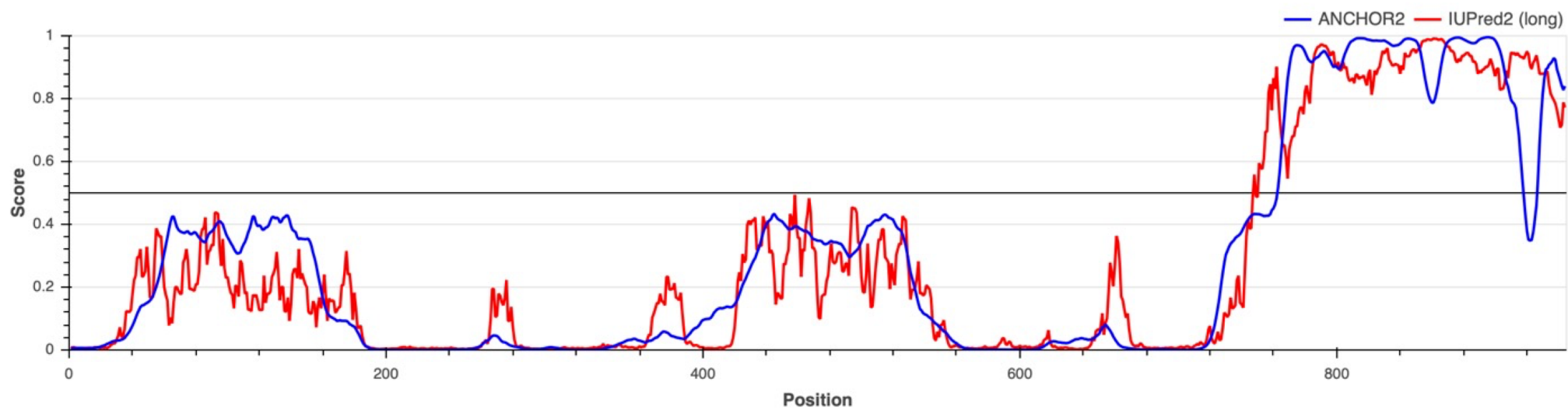

b.

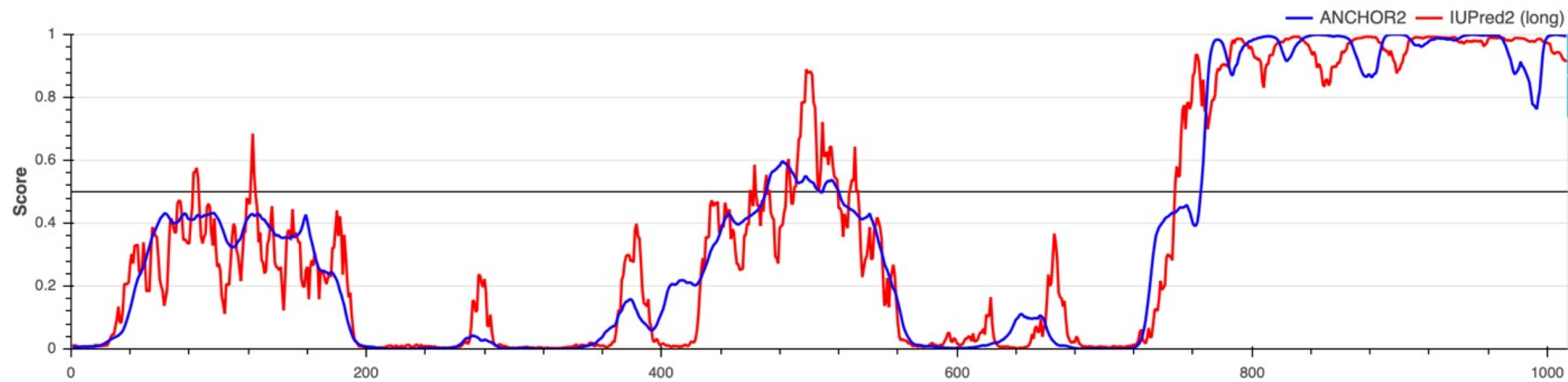

Supplement: Supplemental data [file Suppl_FigS1.pdf]
