# Supplementary material for: Molecular Mechanisms of MmpL3 Function and Inhibition
Source: Microb Drug Resist. 2023 May 4;29(5):190–212. doi: 10.1089/mdr.2021.0424 (PMC10171966; doi:10.1089/mdr.2021.0424)
Supplement: Supplemental data [file Suppl_TableS1.pdf]

Evidence	SQ109	TBL-140	HC2178	E11	E1	AU1235	HC2169	HC2138	P1	CCI7967	BM212	Rimonabant	THPP-1	NITD	ICA-2	BMC/EJMCh	HC2099	HC2183	A1	HC2184	CRS400143 CRS400226	HC2091	HC2060	HC2149	HC2134	GSK	PIPD-1	Spiro	DA-5	C215	SIMBL	DA-8	HC2032	Q1	GSK2623870A	GSK2043267A
Resistant Mutants	+	+	+	+	+	+	+	+	+	+	+	+	+	+	+	+	+	+	+	+	+	+	+	+	+	+	+	+	+	+	+	+	+	+	+	+
TMM/TDM Experiments	+	+	+	+		+	+	+			+	+	+	+	+	+	+	+		+	+	+	+	+	+		+	+	+	+	+	+	+			
Spheroplast Assay	-			+		+					+																+				+					
NORTH Probe Assay	+		+			+	+	+			+		+	+			+	+		+		+	+	+	+					+			+			
SPR or BLI	+					+							+	+																						
Co-Cyrstals	+					+						+		+														+								
PMF Disruption	+	+	+	+		-	-	+			+	+	+	-			-	-		+		-	-	+	-				+	+	+	+	+			

Supplemental Table 1
